# Preoperative predictive factors for intensive care unit admission after pulmonary resection*

**DOI:** 10.1590/S1806-37132015000100005

**Published:** 2015

**Authors:** Liana Pinheiro, Ilka Lopes Santoro, João Aléssio Juliano Perfeito, Meyer Izbicki, Roberta Pulcheri Ramos, Sonia Maria Faresin

**Affiliations:** Federal University of São Paulo, Paulista School of Medicine, São Paulo, Brazil. Department of Pulmonology, Universidade Federal de São Paulo/Escola Paulista de Medicina - UNIFESP-EPM, Federal University of São Paulo Paulista School of Medicine - São Paulo, Brazil; Federal University of São Paulo, Paulista School of Medicine, São Paulo, Brazil. Department of Pulmonology, Universidade Federal de São Paulo/Escola Paulista de Medicina - UNIFESP-EPM, Federal University of São Paulo Paulista School of Medicine - São Paulo, Brazil; Federal University of São Paulo, São Paulo, Brazil, Deputy Dean for Undergraduate Programs. Universidade Federal de São Paulo - UNIFESP, Federal University of São Paulo - São Paulo, Brazil; Federal University of São Paulo, Paulista School of Medicine, São Paulo, Brazil. Department of Pulmonology, Universidade Federal de São Paulo/Escola Paulista de Medicina - UNIFESP-EPM, Federal University of São Paulo Paulista School of Medicine - São Paulo, Brazil; Federal University of São Paulo, Paulista School of Medicine, São Paulo, Brazil. Department of Pulmonology, Universidade Federal de São Paulo/Escola Paulista de Medicina - UNIFESP-EPM, Federal University of São Paulo Paulista School of Medicine - São Paulo, Brazil; Federal University of São Paulo, Paulista School of Medicine, São Paulo, Brazil. Department of Pulmonology, Universidade Federal de São Paulo/Escola Paulista de Medicina - UNIFESP-EPM, Federal University of São Paulo Paulista School of Medicine - São Paulo, Brazil

**Keywords:** Thoracic surgery, Risk factors, Intensive care units

## Abstract

**Objective:**

To determine whether the use of a set of preoperative variables can predict the need for postoperative ICU admission.

**Methods:**

This was a prospective observational cohort study of 120 patients undergoing elective pulmonary resection between July of 2009 and April of 2012. Prediction of ICU admission was based on the presence of one or more of the following preoperative characteristics: predicted pneumonectomy; severe/very severe COPD; severe restrictive lung disease; FEV_1_ or DLCO predicted to be < 40% postoperatively; SpO_2_ on room air at rest < 90%; need for cardiac monitoring as a precautionary measure; or American Society of Anesthesiologists physical status ≥ 3. The gold standard for mandatory admission to the ICU was based on the presence of one or more of the following postoperative characteristics: maintenance of mechanical ventilation or reintubation; acute respiratory failure or need for noninvasive ventilation; hemodynamic instability or shock; intraoperative or immediate postoperative complications (clinical or surgical); or a recommendation by the anesthesiologist or surgeon to continue treatment in the ICU.

**Results:**

Among the 120 patients evaluated, 24 (20.0%) were predicted to require ICU admission, and ICU admission was considered mandatory in 16 (66.6%) of those 24. In contrast, among the 96 patients for whom ICU admission was not predicted, it was required in 14 (14.5%). The use of the criteria for predicting ICU admission showed good accuracy (81.6%), sensitivity of 53.3%, specificity of 91%, positive predictive value of 66.6%, and negative predictive value of 85.4%.

**Conclusions:**

The use of preoperative criteria for predicting the need for ICU admission after elective pulmonary resection is feasible and can reduce the number of patients staying in the ICU only for monitoring.

## Abstract (pt)

**Objetivo:**

Avaliar se a utilização de um conjunto de variáveis pré-operatórias é capaz de antever a necessidade de internação em UTI no pós-operatório.

**Métodos:**

Estudo de coorte observacional prospectivo, com 120 pacientes submetidos à ressecção pulmonar eletiva entre julho de 2009 e abril de 2012. A previsão de indicação de internação em UTI indicação foi baseada na presença de uma ou mais das seguintes condições pré-operatórias: previsão de pneumonectomia; DPOC grave/muito grave; doença restritiva grave; VEF_1_ ou DLCO previstos para o pós-operatório < 40% do previsto; SpO_2_ em repouso e ar ambiente < 90%; necessidade de monitorização cardíaca profilática; classificação da *American Society of Anesthesiologists* ≥ 3. O padrão ouro para internação justificada em UTI foi baseado na presença de uma ou mais das seguintes condições pós-operatórias: manutenção de ventilação mecânica ou reintubação; insuficiência respiratória aguda ou necessidade de ventilação não invasiva; instabilidade hemodinâmica ou choque; intercorrências intraoperatórias ou no pós-operatório imediato (cirúrgicas ou clínicas); indicação do anestesiologista ou cirurgião para a manutenção de tratamento na UTI.

**Resultados:**

Dos 120 pacientes avaliados, houve previsão de necessidade de internação em UTI em 24 (20,0%), sendo essa considerada justificada em 16 deles (66,6%) desses 24, ao passo que dos 96 pacientes sem previsão de necessidade de internação em UTI, essa foi necessária em 14 (14,5%). A utilização dos critérios preditivos para a internação em UTI mostrou boa acurácia (81,6%), sensibilidade de 53,3%, especificidade de 91%, valor preditivo positivo de 66,6% e valor preditivo negativo de 85,4%.

**Conclusões:**

A utilização de critérios pré-operatórios para a indicação de internação em UTI após ressecção pulmonar eletiva é factível e é capaz de reduzir o número de pacientes que aí permanecem apenas para vigilância.

## Introduction

According to the 1997 American Thoracic Society^(^ 1 ^)^ statement regarding ICU allocation decisions, the primary goal of the ICU is "to preserve meaningful human life by protecting and sustaining patients in a caring manner when they are threatened by an acute critical illness or injury or as a consequence of medical or surgical therapy". The 1999 update^(^ 2 ^)^ adds that the ICU serves to monitor and care for patients with potentially severe physiological instability requiring technical and/or artificial life support.

The European Respiratory Society and the European Society of Thoracic Surgeons^(^ 3 ^)^ do not recommend systematic ICU admission after thoracotomy. Patients who are estimated to be at low risk for complications should be referred to a dedicated thoracic surgery unit. Patients with reduced cardiopulmonary reserve undergoing complex resection and patients who are estimated to be at moderate to high risk for complications should be referred to a high-dependency unit, if available, whereas patients requiring support for organ failure should be admitted to the ICU.

It is known that among patients admitted to the ICU after surgery, only a minority develops acute complications requiring immediate intervention; the majority is referred to the ICU for "surveillance" of possible deterioration of their clinical condition or simply for monitoring.^(^ 4 ^-^ 6 ^)^ It should be noted that patients referred to the ICU only for monitoring may experience unfavorable outcomes, such as increased stress due to the environment as well as to sleep and family deprivation. In addition, there is a substantial increase in hospital costs.^(^ 7 ^)^

Thoracic surgery always results in pulmonary dysfunction, and depending on the degree of impairment, there can be difficulty in extubating the patient at the end of the surgical procedure and need for prolonged mechanical ventilation. In addition, many surgical candidates present with comorbidities and/or compromised cardiopulmonary reserve, which makes them more susceptible to developing perioperative complications.^(^ 2 ^,^ 3 ^)^ However, the question regarding the proportion of these patients who would benefit from the ICU setting in the immediate postoperative period also remains unanswered.

Few studies have bothered to determine prognostic factors associated with the need for ICU admission,^(^ 8 ^-^ 11 ^)^ and in the literature there is no consensus regarding indications for ICU admission after pulmonary resection. Therefore, we decided to design a study to investigate whether the use of a set of preoperative variables can predict the need for immediate postoperative ICU admission.

## Methods

A prospective observational cohort study of patients referred to the outpatient preoperative evaluation clinic of the Department of Pulmonology of the *Universidade Federal de São Paulo* (UNIFESP, Federal University of São Paulo) was carried out between July of 2009 and April of 2012. This study was approved by the UNIFESP Research Ethics Committee (Ruling no. 410/09).

We included patients over 18 years of age undergoing elective pulmonary resection, with diagnosed or suspected benign or malignant disease. The exclusion criteria were as follows: incomplete preoperative evaluation; one concomitant surgical procedure in addition to pulmonary resection; parenchymal-sparing procedures; and preoperative or intraoperative death.

After giving written informed consent, participants underwent a preoperative evaluation consisting of clinical assessment and physical examination, with the use of a structured form. Pulmonary function was assessed by using the modified algorithm presented in the American College of Chest Physicians (ACCP) guidelines,^(^ 12 ^)^ with the following tests: pre- and post-bronchodilator spirometry; pulse oximetry; DLCO measurement; pH measurement and arterial blood gas analysis; cardiopulmonary exercise testing; and pulmonary perfusion mapping. On completing the evaluation, patients were categorized as being at high or acceptable risk.

All patients were operated on by the same team of thoracic surgeons and were referred to the ICU of the Pulmonology Section in the immediate postoperative period, being subsequently transferred to the thoracic surgery ward. They were prescribed epidural or intravenous analgesia by the pain team and received physiotherapy until they were discharged.

Preoperative variables collected included age, gender, surgical disease diagnosis, proposed surgery, smoking, respiratory symptoms, comorbidities, Charlson comorbidity index,^(^ 13 ^)^ and American Society of Anesthesiologists (ASA) physical status,^(^ 14 ^)^ as well as baseline and predicted postoperative (ppo) values for FVC, FEV_1_, FEV_1_/FVC, DLCO, and maximal oxygen uptake.

Intraoperative variables analyzed included anesthesia time, procedure performed, number of segments resected, and surgical and clinical complications.

**Chart 1 -:**
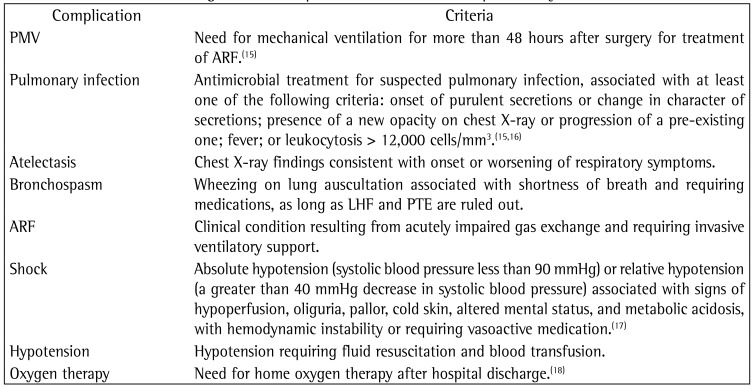
Criteria for the diagnosis of complications observed after pulmonary resection.(15-18) PMV: prolonged mechanical ventilation; ARF: acute respiratory failure; LHF: left heart failure, and PTE: pulmonary thromboembolism.

Postoperative variables analyzed included pulmonary complications (prolonged mechanical ventilation, acute respiratory failure [ARF], pulmonary infection, atelectasis, bronchospasm, and oxygen therapy at discharge); hemodynamic complications (shock, hypotension, cardiac arrhythmia, heart failure, and hypertensive crisis); and surgical complications (bronchial fistula, air leak for more than seven days, bleeding requiring transfusion or reoperation, pneumothorax, and empyema; Chart 1).^(^ 15 ^-^ 18 ^)^

Prediction of the need for ICU admission was based on the presence of one or more of the following preoperative characteristics: planned pneumonectomy; severe/very severe COPD (FEV_1_/FVC < 0.7 and FEV_1_ < 50% predicted); severe restrictive lung disease (FVC < 50% predicted); ppo-FEV_1_ or ppo-DLCO < 40%; SpO_2_ on room air at rest < 90%; need for cardiac monitoring as a precautionary measure; or ASA physical status ≥ 3.

The gold standard for mandatory admission to the ICU was based on the presence of one or more of the following postoperative characteristics: need for maintenance of invasive mechanical ventilation after surgery or for reintubation; ARF or need for noninvasive positive pressure ventilation; hypotension with hemodynamic instability or associated with signs of shock, requiring blood transfusion as well as fluid resuscitation; unresolved intraoperative or immediate postoperative complications (clinical or surgical); or a recommendation by the anesthesiologist or surgeon to continue treatment in the ICU.

## Statistical analysis

Categorical variables were summarized as absolute and relative frequencies (percentages). Numerical variable data were expressed as mean, standard deviation, median, and interquartile range. Diagnostic performance of the ICU admission prediction model was tested by calculating sensitivity, specificity, positive predictive value, negative predictive value, and accuracy, and was measured against the gold standard for mandatory ICU admission. All data were tabulated using Microsoft^(r)^ Excel 2007. For statistical calculations, we used the Statistical Package for the Social Sciences for Windows, version 19.0 (IBM SPSS Statistics, IBM Corp., Armonk, NY, USA).

## Results

**Figure 1 -:**
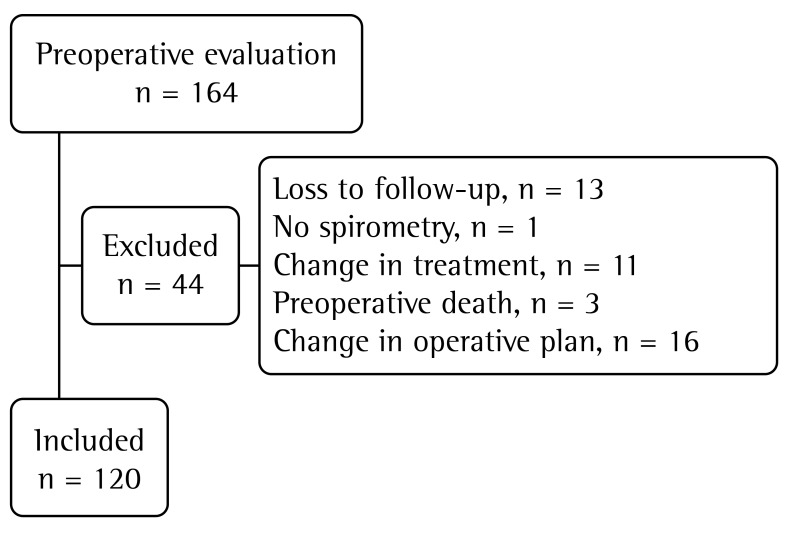
Flowchart of patient selection.

Figure 1 depicts the flowchart of patient selection. The clinical and functional characteristics of the 120 patients included in the study are shown in Table 1.

**Table 1 -:** Clinical, functional, and histopathological characteristics of the 120 patients undergoing pulmonary resection.a

Characteristic	Result
Male gender	65 (54.2)
Age (years)	56.2 ± 12.3
Number of respiratory symptoms^b^	2 (0-3)
Number of comorbidities^b^	2 (1-3)
Charlson index^b^	3 (2-4)
Smoker	75 (62.5)
ASA physical status	
1	4 (3.3)
2	111 (92.5)
3	5 (4.2)
Functional parameter	
FVC	88.4 ± 17.6
FEV_1_	82.3 ± 19.2
FEV_1_/FVC	0.75 ± 0.1
ppo-FEV_1_	72.4 ± 19.3
DLCO	64.4 ± 19.0
ppo-DLCO	53.4 ± 15.2
Histopathological diagnosis	
Benign disease	41 (34.2)
Malignant disease	79 (65.8)
Primary lung cancer	52 (43.3)
Metastatic cancer	27 (22.5)

ASA: American Society of Anesthesiologists; and ppo: predicted postoperative.

a Values expressed as n (%) or as mean ± SD, except where otherwise indicated.

b Values expressed as median (interquartile range).

DLCO was measured in 31 patients (25.8%), and its mean ± SD was 64.4 ± 19% predicted. Maximal oxygen consumption was determined in 4 patients who underwent cardiopulmonary exercise testing, and its mean was 21 mL • kg^-1^ • min^-1^ or 74.5% predicted.

The mean anesthesia time was 6.1 ± 1.8 hours. The surgical procedures performed were pneumonectomy (in 16 patients; 13.4%); lobectomy (in 58; 48.3%); bilobectomy (in 1; 0.8%); trisegmentectomy (in 2; 1.6%); segmentectomy (in 16; 13.4%); and other minor operations (in 27; 22.5%).

Pathological examination of the surgical specimens revealed benign disease in 41 patients (34.1%) and malignant disease in 79 (65.9 %; Table 1).

Thirty-nine patients (32.5%) had a total of 89 complications. Of those, 64 (72%) were clinical complications and 25 (28%) were surgical complications. Hypotension requiring fluid resuscitation and blood transfusion or shock of various etiologies was the most common clinical complication (21; 23.7%), followed by pulmonary infection (15; 16.9%), ARF and/or need for mechanical ventilation after surgery (10; 11.2%), arrhythmia (7; 7.9%), and bronchospasm (3; 3.4%). There were two episodes (2.2%) of each of the following complications: atelectasis; acute pulmonary edema; need for oxygen therapy at hospital discharge; and hypertensive crisis. Bleeding was the most common surgical complication (11; 12.3%), followed by bronchial fistula (5; 5.6%), empyema (4; 4.5%), prolonged air leak (3; 3.4%), and pneumothorax (2; 2.2%).

The 30-day mortality rate was 2.5% (3/120), and three deaths were due to septic shock from pulmonary infection, which, in 2 cases, was preceded by a bronchial fistula. The 3 patients in question had a diagnosis of bronchiectasis with aspergilloma. Another 4 deaths occurred after 30 days, but during the same hospital stay: 2 occurred during an operation for bronchiectasis and 2 occurred during an operation for cancer.

Among the 120 patients evaluated, 24 (20.0%) were predicted to require ICU admission, and the clinical criteria for this prediction are shown in Table 2.

**Table 2 -:** Factors for predicting the need for ICU admission in 24 patients undergoing pulmonary resection.

Reason	n (%)
Pneumonectomy	13 (10.8)
ASA physical status 3	5 (4.2)
Severe COPD	3 (2.5)
ppo-FEV_1_ or ppo-DLCO < 40%	3 (2.5)
Cardiac condition	2 (1.7)
SpO_2_ < 90%	2 (1.7)
Severe RLD	1 (0.8)

ASA: American Society of Anesthesiologists; ppo: predicted postoperative; and RLD: restrictive lung disease.

Among those 24 cases, there were 16 true positives and 8 false positives. Of those 8 false positives, 1 underwent surgical treatment of bleeding bronchiectasis, had restrictive lung disease, and had a ppo-DLCO of 33%, and 1 had severe COPD, with a preoperative FEV_1_ of 45% predicted and an SpO_2_ of 88%. In the remaining 6, the proposed surgical procedure was pneumonectomy. All false positives were discharged from the ICU in the morning after the procedure (Table 3).

**Table 3 -:** Distribution of the 120 patients undergoing pulmonary resection by ICU admission status.

Predicted ICU admission, n (%)	Mandatory ICU admission, n (%)		Total
	Yes	No	
Yes	16 (67)	8 (33)	24
No	14 (15)	82 (85)	96
Total	30	90	120

Among the 96 patients for whom ICU admission was not predicted, there were 96 true negatives and 14 false negatives. Those 14 false negatives were ASA physical status 2, had a mean age of 60 years, and had a mean ppo-FEV_1_ of 70%; in addition, the proposed surgical procedure was not pneumonectomy (Table 3).

The accuracy of the ICU admission prediction model was 81.6% (98/120), with sensitivity of 53.3%, specificity of 91%, positive predictive value of 66.6%, and negative predictive value of 85.4%.

Analysis of the false negative subgroup revealed that, in 2 cases, the operation was converted from lobectomy to pneumonectomy intraoperatively; 2 patients developed arrhythmia, which was promptly corrected, and 12 patients had shock or hypotension requiring transfusion, the shock being caused by intraoperative or immediate postoperative bleeding in 10 (Table 4).

**Table 4 -:** Characteristics of the patients for whom ICU admission was not predicted but who required it (mandatory ICU admission).

Patient	Age	ASA physical status	Disease	Resection	Reason for ICU admission
1	66	2	SCC; COPD; arrhythmia; SAH	Left lung	Arrhythmia
2	63	2	SCC	Left lung	Bleeding; HVC; APE; MV
3	58	2	DM; SAH; cystadenoma	LUL	Bleeding; HVC; MV; bronchial fistula
4	70	2	Adenoca; RA; vesical tumor	RUL/seg. VI	Bleeding; hypotension
5	71	2	SCC; neolarynx	RUL	ARF; MV; shock
6	72	2	SCC	LLL/lingula	Bleeding; HVC; MV
7	77	2	Nodule in the RLL	Nodule resection	Hypertensive crisis; arrhythmia
8	54	2	Nodule in the LSD	RUL	Bleeding; hypotension
9	63	2	Metastasis	Metastasectomy	Bleeding; HVC; APE
10	28	2	BE	RUL	Bleeding; hypotension
11	34	2	BE	RUL	Bleeding; HVC; MV
12	62	2	BE	ML/seg. VI	Shock
13	56	2	BE/aspergilloma	RUL	Bronchial fistula; MV; hemothorax; HVC
14	63	2	BE/aspergilloma	LUL	Bleeding; HVC

ASA: American Society of Anesthesiologists; SCC: squamous cell carcinoma; SAH: systemic arterial hypertension; HVC: hypovolemic shock; APE: acute pulmonary edema; MV: mechanical ventilation; DM: diabetes mellitus; LUL: left upper lobe; RA: rheumatoid arthritis; RUL: right upper lobe; seg.: segment; ARF: acute respiratory failure; LLL: left lower lobe; RLL: right lower lobe; BE: bronchiectasis; and ML: middle lobe.

## Discussion

Studies on criteria for predicting ICU admission after pulmonary resection are few in number and have included mainly patients with lung cancer. ^(^ 7 ^,^ 10 ^,^ 11 ^)^ When planning this study, we decided to include patients with benign diseases as well, because, in developing countries, surgical procedures for the treatment of bronchiectasis and other post-infectious pulmonary sequelae are still very common. Benign diseases are known to be more prevalent in patients younger than those presenting with cancer, but this does not make the rates of postoperative morbidity (18 to 46%) and mortality (zero to 26.3%) any lower.^(^ 19 ^-^ 21 ^)^

In 2008, Brunelli et al.^(^ 10 ^)^ developed and validated the first risk scale for predicting the need for ICU admission after pulmonary resection, principally for patients with lung cancer. Of the 1,297 participants, 82 (6.3%) required ICU admission, and, using the logistic regression model, those authors found that the independent predictors of need for ICU admission were age over 65 years, ppo-FEV_1_ < 65%, ppo-DLCO < 50%, cardiac comorbidities, and pneumonectomy.

Okiror et al.^(^ 11 ^)^ performed external validation of the scale developed in aforementioned study and concluded that the scale of Brunelli et al. had a moderate discriminatory power for predicting the need for ICU admission. However, the criteria used in the validation study were not the same as those of the original study (emergency ICU admission vs. elective ICU admission), which affected the results of the validation study.

Therefore, when analyzing studies of prognostic risk models, it is necessary to consider the population they apply to, and when it comes to validation studies, it is necessary to determine whether the circumstances under which they were carried out can be superimposed to those of the original study. However, it is important to bear in mind that most health care facilities do not have the technological means to run sophisticated models, nor do they follow strict inclusion protocols.

What could then be done in this context?

Pieretti et al.^(^ 9 ^)^ used a set of pre-established criteria for predicting the need for ICU admission and obtained satisfactory results. Therefore, at our facility-i.e., a university hospital-we decided to determine whether the use of a set of preoperative clinical variables for predicting the need for ICU admission would be able to accomplish this goal.

The choice of the clinical criteria used for predicting the need for ICU admission was based on data in the literature and on our daily clinical practice. Pneumonectomy accounts for the highest morbidity and mortality rates among the various possible resections.^(^ 20 ^-^ 23 ^)^ ASA physical status 3 means that the patient has severe systemic disease resulting in functional limitation in activities of daily living.^(^ 14 ^,^ 24 ^-^ 26 ^)^ Values of < 50% predicted for FEV_1_ and FVC, as determined by spirometry, are associated with severe or very severe disease. In a scenario of a patient with reduced functional reserve, who will undergo removal of nonfunctioning parenchyma, the use of ppo-FEV_1_ or ppo-DLCO for predicting the need for postoperative ICU admission corrects this distortion, to some extent, and a threshold of 40% has been established by the ACCP guidelines. ^(^ 12 ^)^ An SpO_2_ on room air at rest < 90% indicates reduced functional reserve and worsening of gas exchange during the removal of still functioning parenchyma. ^(^ 28 ^,^ 29 ^)^ Cardiac monitoring as a precautionary measure, the need for which is defined by the cardiologist, is recommended in patients with reduced cardiovascular reserve.^(^ 12 ^)^

Patients were predicted to require ICU admission because there was a major surgical risk factor or because their clinical status was compromised either by reduced cardiopulmonary reserve or by comorbidities. However, the prediction was incorrect in 8 patients (false positives), and the predictive factors were ppo-DLCO < 40%, severe COPD with an SpO_2_ on room air of 88%, and planned pneumonectomy. This finding could signal that, even in large operations, if there are no other risk factors, the patient could be sent to an intermediate care unit and not necessarily to the ICU.

Among the 96 patients for whom the need for ICU admission was not predicted, the non-prediction was incorrect in 14 (false negatives), a situation that is a greater cause for concern than is predicting the need for ICU admission when that is not the case, if the error endangers the postoperative course of such patients. All the complications that justified the patient staying in the ICU occurred during or soon after the surgical procedure, namely: shock and/or hypotension requiring blood transfusion as well as fluid resuscitation; and intraoperative conversion from lobectomy to pneumonectomy. And this scenario could not have been predicted preoperatively.

Although these complications ended up reducing the sensitivity of the criteria used, which was 53.3%, the specificity and negative predictive value of the criteria were encouraging, 91% and 85.4%, respectively, and their accuracy was 81.7%.

These findings make us think that prediction of the need for ICU admission after pulmonary resection should consider the possibility of major bleeding and not only the size of the operation or the clinical status of the patient. Most studies addressing prediction of the need for ICU admission in pulmonary resection candidates have focused mainly on patients with malignant lung diseases,^(^ 7 ^-^ 11 ^)^ and our study showed that, among patients with bronchiectasis, the rate of intraoperative bleeding was more than twice as high as that among patients with lung cancer.

The major limitation of our study is its sample size, which did not allow the internal validation of the study. The inclusion of patients from a single facility can limit the size of the population sample and preclude the generalization of results. However, it has several advantages: it is convenient for the population that seeks our services; all surgical procedures are performed by the same surgical team; preoperative clinical assessment is also performed by the same team and always in the same way, regardless of whether that is a clinical study or that is simply health care; and the care provided in the ICU and the follow-up care by physiotherapists and by the pain team of the hospital are consistent with our peculiarities and difficulties. Another minimal limitation is the 53.3% sensitivity of the model, a value that reduces its reliability in predicting the need for ICU admission after pulmonary resection. However, this model ensures high specificity and high negative predictive value in predicting which patients will not require ICU admission.

In conclusion, the use of composite measures for predicting the need for ICU admission after pulmonary resection is feasible and accurate, and since this model uses clinical variables that do not require high technology (planned pneumonectomy; severe COPD; severe restrictive lung disease; ppo-FEV_1_ or ppo-DLCO < 40%; SpO_2_ on room air at rest < 90%; need for cardiac monitoring as a precautionary measure; ASA physical status ≥ 3), it may have wide applicability in daily clinical practice.
